# Early changes in cardiovascular structure and function in adolescents with type 1 diabetes

**DOI:** 10.1186/s12933-016-0351-3

**Published:** 2016-02-16

**Authors:** Timothy J. Bradley, Cameron Slorach, Farid H. Mahmud, David B. Dunger, John Deanfield, Livia Deda, Yesmino Elia, Ronnie L. H. Har, Wei Hui, Rahim Moineddin, Heather N. Reich, James W. Scholey, Luc Mertens, Etienne Sochett, David Z. I. Cherney

**Affiliations:** Department of Paediatrics, Division of Cardiology, Department of Paediatrics, The Hospital for Sick Children, University of Toronto, Toronto, Canada; Department of Paediatrics, Division of Endocrinology, JDRF-Canadian Clinical Trial Network (JDRF-CCTN) Sick Kids Multicenter Clinical Trial Center, The Hospital for Sick Children, University of Toronto, Toronto, Canada; Department of Pediatrics, University of Cambridge, Cambridge, UK; University College Hospital, London, UK; Heart Hospital and Great Ormond Street Hospital, London, UK; Department of Family and Community Medicine, University of Toronto, Toronto, Canada; Department of Medicine, Division of Nephrology, University Health Network, Toronto General Hospital, University of Toronto, 585 University Avenue, 8 N-845, Toronto, ON M5G 2N2 Canada

**Keywords:** Adolescents, Type 1 diabetes, Echocardiography, Myocardial contraction

## Abstract

**Background:**

Children with type 1 diabetes (T1D) are at higher risk of early adult-onset cardiovascular disease. We assessed cardiovascular structure and function in adolescents with T1D compared with healthy controls and the relationships between peripheral vascular function and myocardial parameters.

**Methods and results:**

199 T1D [14.4 ± 1.6 years, diabetes duration 6.2 (2.0–12.8) years] and 178 controls (14.4 ± 2.1 years) completed endothelial function by flow mediated vasodilatation (FMD), arterial stiffness using pulse wave velocity (PWV) along with M-mode, pulse wave and tissue Doppler, and myocardial deformation echocardiographic imaging. Systolic (113 ± 10 vs. 110 ± 9 mmHg; p = 0.0005) and diastolic (62 ± 7 vs. 58 ± 7 mmHg; p < 0.0001) blood pressures, carotid femoral PWV and endothelial dysfunction measurements were increased in T1D compared with controls. Systolic and diastolic left ventricular dimensions and function by M-mode and pulse wave Doppler assessment were not significantly different. Mitral valve lateral e’ (17.6 ± 2.6 vs. 18.6 ± 2.6 cm/s; p < 0.001) and a’ (5.4 ± 1.1 vs. 5.9 ± 1.1 cm/s; p < 0.001) myocardial velocities were decreased and E/e’ (7.3 ± 1.2 vs. 6.7 ± 1.3; p = 0.0003) increased in T1D. Left ventricular mid circumferential strain (−20.4 ± 2.3 vs. −19.5 ± 1.7 %; p < 0.001) was higher, whereas global longitudinal strain was lower (−19.0 ± 1.9 vs. −19.8 ± 1.5 % p < 0.001) in T1D.

**Conclusions:**

Adolescents with T1D exhibit early changes in blood pressure, peripheral vascular function and left ventricular myocardial deformation indices with a shift from longitudinal to circumferential shortening. Longitudinal follow-up of these changes in ongoing prospective trials may allow detection of those most at risk for cardiovascular abnormalities including hypertension that could preferentially benefit from early therapeutic interventions.

## Background

The development of abnormal ventricular function in diabetes is multifactorial and has been attributed to metabolic disturbances, renal impairment, myocardial fibrosis, small vessel disease, cardiac autonomic neuropathy, and insulin resistance [1, 2]. In adults longer duration of diabetes is associated with impairment of systolic and diastolic function [3]. Glycemic changes and lipid abnormalities are associated with early vascular changes in children with T1D [4], but less is known in regard to how these metabolic disturbances affect myocardial structure and function. Given that adolescence is a critical period associated with onset and progression of vascular complications and poor glycemic control, it is important to evaluate central cardiac and peripheral vascular changes.

Diabetes has long been known to be a major risk factor for the development of cardiovascular disease [5]. Consequently, children with type 1 diabetes (T1D) are at higher risk of early adult-onset cardiovascular disease [6]. In adults increased morbidity and mortality have been observed due to coronary, cerebrovascular, and peripheral arterial disease [7], and early vascular changes have been shown to be present from childhood [8, 9]. To date, however, little is known about the evolution of early cardiac structural and functional abnormalities in children with T1D, as previous echocardiographic studies have shown variable differences in left ventricular (LV) geometry, mass and function [10–15]. More recently, myocardial deformation imaging has been shown to detect early changes in LV myocardial mechanics in children and adolescents with T1D prior to changes in ejection fraction [16].

Accordingly, our aim was to evaluate cardiovascular structure and function in adolescents with T1D compared with healthy controls and to assess the relationships between these peripheral vascular and myocardial parameters with clinical parameters in patients with T1D.

## Methods

### Study population

Patients were recruited from the longitudinal, observational, non-interventional arm of the adolescent type 1 diabetes cardio-renal intervention trial (AdDIT) from clinical sites in the Greater Toronto Area. In brief, the non-randomized low-risk arm of AdDIT is a 4-year observational/natural history study, following adolescents at low and middle risk of developing microalbuminuria (EudraCT Number: 2007-001039-72) [17]. High-risk adolescents are recruited into the AdDIT interventional study (http://www.clinicaltrials.gov/ct2/show/NCT01581476), which was designed to examine the effect of angiotensin converting enzyme inhibitors and statins on renal, retinal and cardiovascular endpoints. Our study did not include participants involved in the intervention trial; however, as an ancillary component of non-randomized low-risk arm of AdDIT we also included high-risk subjects who chose not to enter the AdDIT intervention study. All analyses in this manuscript were performed using biological specimens and data collected from subjects enrolled in the observational and ancillary arm of AdDIT, specifically baseline data from the study obtained at Greater Toronto Area sites.

Patients with uncomplicated T1D were recruited from endocrinology clinics at The Hospital for Sick Children, Credit Valley Hospital and Markham–Stouffville in the Greater Toronto Area (Ontario, Canada), and inclusion/exclusion criteria have been described elsewhere [18]. Healthy controls were recruited through local advertisements. The Hospital for Sick Children was the primary site of recruitment and also coordinated recruitment at the secondary sites (REB# 1000012240). The Hospital for Sick Children Research Ethics Board, Credit Valley Hospital Ethics Forum and Markham–Stouffville Research Ethics Board approved the protocol and the consent procedure. Written informed consent was obtained from the legal guardian/next of kin/caretakers of minors aged 15 and younger, while the minors provided assent. All subjects, aged 16 and older with capacity to understand the study information, gave complete written and informed consent to participate in the study.

Control subjects were recruited as similar aged healthy volunteers, who were not on any vasoactive medications, had no previous history of familial hyperlipidemia, diabetes, obesity, hypertension, or any other significant cardiac, renal or systemic disease and normal cardiac anatomy and function by screening echocardiogram [18].

### Clinical assessment

For adolescents with T1D, data on chronological age, age at diabetes onset, and duration of diabetes were collected. For all subjects, height was measured by a wall-mounted stadiometer and weight by electronic scales and resting heart rate and right brachial blood pressure was measured using an age-appropriate cuff and averaging three readings with an automated DINAMAP^®^ sphygmomanometer (Critikon, Tampa, Florida, USA). Systolic and diastolic blood pressures were also converted to z-scores for age, sex and height [19]. For all T1D and a subgroup of 65 controls that underwent the same baseline clinical assessment, fasting blood samples were collected for blood glucose, HbA1c measurements and lipid profiles [total cholesterol, HDL (high-density lipoprotein) cholesterol, LDL (low-density lipoprotein) cholesterol and triglycerides] and serum cystatin C was used to estimate glomerular filtration rate, as previously described [20].

### Echocardiographic assessment

Using a Vivid 7 ultrasound system (GE, General Electric Corp, Wisconsin, USA) a standardized functional imaging protocol was performed according to published guidelines [21], and using methods as previously described by our group [22–24].

LV end-diastolic and end-systolic dimensions and thicknesses were measured using M-mode in the parasternal short-axis view. LV mass indexed to body surface area (BSA), shortening fraction, ejection fraction and heart rate-corrected velocity of circumferential fiber shortening were calculated.

Pulse wave Doppler images were acquired over three cardiac cycles with care taken to ensure parallel cursor alignment to flow and to adjust scale and filters to optimize the spectral Doppler tracings. Mitral valve (MV) E and A wave peak velocities, E-wave deceleration times and A wave duration were measured in the apical four-chamber view with a 2 mm sample volume placed at the tips of the MV leaflets. Isovolumic relaxation time was measured in the apical five-chamber view. Pulmonary vein S and D wave velocities were measured in the right pulmonary vein. LV myocardial performance index was calculated ${{\left( {{\text{MV closure to opening time}} - {\text{LV ejection time}}} \right)} \mathord{\left/ {\vphantom {{\left( {{\text{MV closure to opening time}} - {\text{LV ejection time}}} \right)} {\text{LV ejection time}}}} \right. \kern-0pt} {\text{LV ejection time}}}$. MV closure to opening time was measured in the apical four-chamber view, as the time from the cessation of the A wave to the onset of the E wave of MV inflow. LV ejection time was measured in the apical five-chamber view with a 2 mm sample volume placed immediately below the aortic valve annulus, as the time from the onset to cessation of LV outflow. LV stroke volume was estimated using the LV outflow cross-sectional area calculated from the dimension measured in the parasternal long-axis view multiplied by the LV outflow velocity time integral determined from the LV outflow pulse wave Doppler spectral tracing in the apical five-chamber view. Cardiac output was estimated as stroke volume multiplied by heart rate.

Pulse tissue Doppler images were acquired over three cardiac cycles from the apical four-chamber view with a 5 mm sample volume placed just below the lateral and septal MV annulus. MV lateral and septal e’, a’ and s’ tissue velocities were measured and the E/e’-ratios were calculated.

Myocardial deformation was assessed by measuring LV mid circumferential strain, global longitudinal strain, basal and apical rotation and torsion. Grey-scale images acquired at frame rates between 40 and 90 frames per second were optimized for off-line speckle tracking analysis. Images acquired at the basal, mid and apical ventricular levels in the parasternal short-axis view were used for assessment of circumferential strain, rotation and torsion, and in the apical four-chamber view for assessment of longitudinal strain. The grey-scale images were analyzed offline on the EchoPAC software (General Electric, Milwaukee, WI, USA). The endocardial border was manually traced (starting at the mid-septum for short axis images and the basal septum from the apical four chamber images). Tracking was automatically performed and the analysis was accepted after visual inspection and when the software indicated adequate tracking or retraced with manual adjustments. Circumferential strain curves from the mid ventricular level in the parasternal short-axis view were analysed to determine mid circumferential strain. Longitudinal strain curves from the apical four-chamber view were analysed for six segments (basal septum, mid septum, apical septum, apical lateral, mid lateral and basal lateral) to determine the global longitudinal strain. Only image acquisitions with a minimum of four appropriately tracking segments were accepted for analysis. LV rotation was calculated by analysis at the basal and apical levels in the parasternal short-axis and torsion calculated as the peak instantaneous end-systolic difference between apical and basal rotation.

### Assessment of endothelial function and arterial stiffness

Vascular assessments were conducted after 10 min of rest in the supine position, in a 25 °C temperature-controlled, quiet room. Measurements were taken at approximately 0900 h in the fasted state. Peripheral blood pressure was measured in the right brachial artery with an automated DINAMAP^®^ sphygmomanometer (Critikon, Tampa, Florida, USA) [25]. Conduit artery endothelial function was determined by recording diameter changes in the brachial artery in response to increased blood flow generated during reactive hyperemia, flow-mediated dilatation (FMD) [25, 26]. Briefly, the right brachial artery was scanned 2–5 cm above the antecubital fossa using high resolution B-mode vascular ultrasound (Vivid 7 ultrasound machine, 7–15 MHz linear-array transducer). Longitudinal, ECG-gated, end-diastolic images were acquired and the brachial arterial diameter was determined for each image using automatic edge-detection software (Vascular Tools, Coralville, IA). Images were recorded for 1 min before a pressure cuff, around the forearm distal to the elbow, was inflated to >200 mmHg for 5 min. After cuff deflation, the increase in blood flow was measured (reactive hyperemia) along with the change in vessel diameter (endothelium-dependent dilatation), which was measured for a further 5 min. FMD  % changes were defined as the maximal percentage changes in vessel diameter after reactive hyperemia as we have previously described [25, 26]. The variability for repeated measurements of arterial diameters at flow mediated vasodilatation was 0.26 ± 0.01 % (% absolute value of brachial artery at FMD), which is similar to previous report [27].

Arterial stiffness was also assessed using pulse wave velocity (PWV) by sequentially recording ECG-gated right carotid and radial artery waveforms (carotid-radial PWV), as well as right carotid and femoral artery (carotid–femoral PWV) waveforms (SPC-301, Millar Instruments SphygmoCor, AtCor Medical Systems Inc., Sydney, Australia). Two vascular measurements were obtained for each parameter and the average value reported. The use of the SphygmoCor device to assess arterial stiffness parameters has been previously published by our group [28]. Automated blood pressure measurements were obtained using a DINAMAP machine (Critikon, Tampa, Florida) and the average of two values obtained immediately prior to the arterial stiffness assessments are reported.

### Statistical analysis

Data are summarized as mean ± SD unless otherwise specified. Comparisons between groups were performed using χ^2^ or Fisher’s exact test for categorical variables and unpaired Student’s *t* tests or analysis of variance for continuous variables. We used linear regression to assess adjusted relationship between echocardiographic measures and potential explanatory clinical variables adjusting for age, gender, SBP, DBP, HbA1c, ACR, LDL, and BMI. Statistical significance was considered at p < 0.05 and we used the false discovery rate (FDR) method to control for the multiple testing. All statistical analysis was carried out using SAS 9.4 (SAS Institute, Cary, NC, USA).

## Results

### Baseline clinical characteristics

**Fig. 1:**
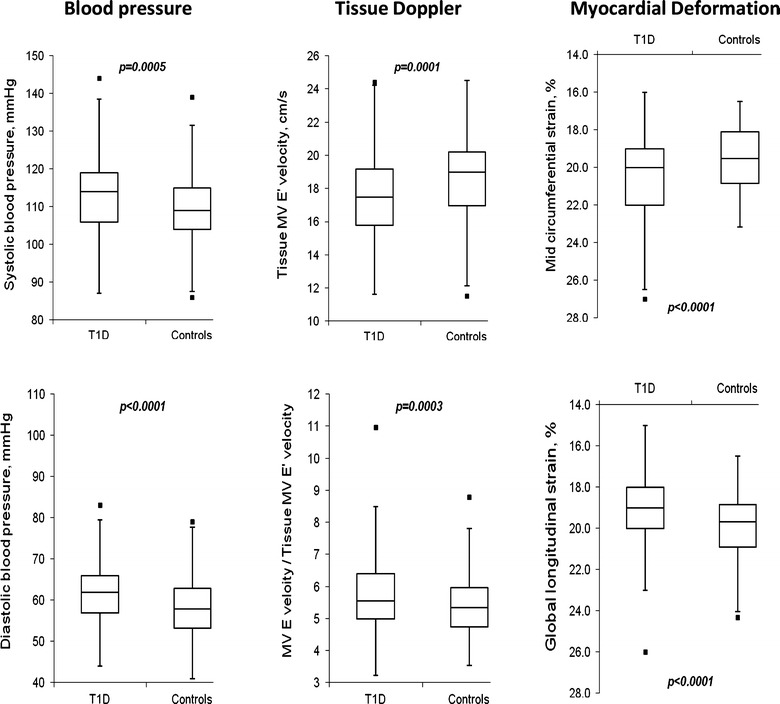
*Box* and *whisker plots* of significant group differences in blood pressure and echocardiographic measurements between adolescents with type 1 diabetes and controls. *Boxes* represent inter-quartile ranges (IQR), the ends of the *whisker* are set at 1.5* IQR above the third quartile and 1.5* IQR below the first quartile, and the minimum or maximum outliers are only shown if outside this range

We compared 199 adolescents with T1D [median disease duration 6.2 (2.0–12.8) years] with all 178 healthy control subjects. These groups were well matched for sex, age and height (see Table 1), but T1D were heavier with larger BSA and body mass index (BMI). T1D had increased systolic and diastolic blood pressures (see Fig. 1), but only diastolic blood pressure remained significantly different when converted to z-scores for height. In the diabetes cohort, more participants were insulin pumper users (Table 1). The proportion of participants who had smoked cigarettes in the past or were current smokers is shown in Table 1 (p = 0.45 for between group difference in rate of smoking in T1D vs. the control group).

**Table 1:** Clinical measurements of adolescents with type 1 diabetes versus all controls

	Type 1 diabetes n = 199	All controls n = 178	p value
Sex (male:female)	98:101	84:94	0.69
Age (years)	14.4 ± 1.6	14.4 ± 2.1	0.80
Height (cm)	163 ± 10	162 ± 11	0.25
Weight (kg)	60.7 ± 14.3	54.7 ± 13.8	<0.0001
BSA (m^2^)	1.65 ± 0.22	1.57 ± 0.23	0.0006
Insulin administration (%)			
Pump	60.8	–	
Multiple injections	30.2	–	
Smoking status *n* (%)			
Never smoked	181 (92.8)	63 (100)	
Smoked few puffs to a whole cigarette in my life	9 (4.6)	0	
Only 2 to 3 cigarettes in my life	1 (0.5)	0	
More than 3, but fewer than 100 cigarettes in my life	1 (0.5)	0	
>100 cigarettes in my life, none in the past month	1 (0.5)	0	
>100 cigarettes in my life, some in the last month but not every day	1 (0.5)	0	
>100 cigarettes in my life, at least 1 cigarette every day during the last month	2 [1]	0	
BMI (kg/m^2^)	22.6 ± 4.3	20.6 ± 3.7	<0.0001
SBP (mmHg)	113 ± 10	110 ± 9	0.0005
SBP *z*-score for height	0.2 ± 0.9	−0.1 ± 0.9	0.0011
DBP (mmHg)	62 ± 7	58 ± 7	<0.0001
DBP *z*-score for height	−0.3 ± 0.6	−0.6 ± 0.7	<0.0001
MBP (mmHg)	77 ± 7	75 ± 7	0.0053
PP (mmHg)	51 ± 9	52 ± 8	0.48
HR (bpm)	66 ± 9	68 ± 12	0.036
Diabetes duration (years)	7.2 ± 3.1	–	−
Glucose (mmol/l)	9.7 ± 4.4	4.7 ± 0.7	<0.0001
HbA1c (%)	0.085 ± 0.012	0.054 ± 0.002	<0.0001
HbA1c mmol/mol	84.9 ± 12.4	53.8 ± 2.4	<0.0001
Cholesterol (mmol/L)	4.3 ± 0.88	4.24 ± 0.83	0.5158
HDL cholesterol (mmol/L)	1.6 ± 0.36	1.46 ± 0.28	0.0004
LDL cholesterol (mmol/L)	2.3 ± 0.71	2.36 ± 0.74	0.6109
Triglycerides (mmol/L)	0.84 ± 0.37	0.93 ± 0.40	0.0945
Creatinine (mmol/L)	54 ± 9	56 ± 11	0.1149
eGFR (mL/min/1.73 m^2^)	113 ± 17	108 ± 17	0.0405

Blood work, smoking status data were available for a subgroup of n = 63 controls

Data are mean ± SD. *BSA* body surface area, *BMI* body mass index, *SBP* systolic blood pressure, *DBP* diastolic blood pressure, *MBP* mean blood pressure, *PP* pulse pressure, *HR* heart rate, *PP* pulse pressure, *HR* heart rate, *HbA1c* haemoglobin A1c, *HDL* high-density lipoprotein, *LDL* low-density lipoprotein cholesterol, *eGFR* estimated glomerular filtration rate

### Endothelial function and arterial stiffness in the T1D and healthy control cohorts

Endothelial function as assessed by FMD was significantly lower in the T1D compared to the healthy control group (6.45 ± 3.15 vs. 7.52 ± 3.20 %, p = 0.0015). For arterial stiffness, carotid-radial PWV was significantly higher in T1D vs. healthy controls (7.28 ± 0.96 vs. 6.89 ± 1.11 m/s, p = 0.0015). Similar trends were seen for carotid-femoral PWV, although differences did not reach significance (5.25 ± 0.75 vs. 5.10 ± 0.87 m/s, p = 0.073).

### Relationships of endothelial function and arterial stiffness with clinical data

Male gender was the only variable that explained a proportion of the difference in FMD, between the T1D and control groups (β = −1.13 ± 0.43, p = 0.0132). For carotid-radial PWV, the variables that explained differences between the T1D and control groups were diastolic blood pressure (β = 0.056 ± 0.010, p = 0.0002) and male gender (β = 0.307 ± 0.123, p = 0.0138).

### Echocardiographic assessment in the T1D and healthy control cohorts

Echocardiographic assessment adjusted for sex, age and BSA to accommodate for any differences in body proportions between the groups are presented in Table 2; Fig. 1. Using M-mode echocardiography, smaller LV end-systolic dimension and higher shortening fraction and ejection fraction were present in T1D compared with controls. Based on pulsed wave Doppler assessment of mitral inflow and pulmonary venous flow, isovolumic relaxation time was higher in T1D vs. control participants, but there were no other significant differences in T1D compared with controls. By pulsed wave tissue Doppler assessment, T1D had significantly lower MV lateral and septal e’ and a’ and septal e’ myocardial velocities and higher E/e’ ratios. By myocardial deformation imaging, T1D had lower LV global longitudinal strain and higher mid circumferential strain, suggesting lower longitudinal, but higher circumferential function. Apical rotation and torsion were both higher in the T1D, while basal rotation did not differ.

**Table 2:** Echocardiographic measurements of adolescents with type 1 diabetes versus all controls

	Type 1 diabetes n = 199	All controls n = 178	p value*
M-mode			
IVS thickness in diastole (cm)	0.74 ± 0.12	0.73 ± 0.12	0.43
LV end-diastolic dimension (cm)	4.69 ± 0.39	4.66 ± 0.43	0.09
LV end-systolic dimension (cm)	2.90 ± 0.32	2.95 ± 0.33	0.0032
LV posterior wall thickness in diastole (cm)	0.66 ± 0.11	0.64 ± 0.11	0.72
LV mass indexed (g/m^2^)	63 ± 13	63 ± 13	0.32
LV shortening fraction (%)	38 ± 4	37 ± 4	0.012
LV ejection fraction (%)	68 ± 5	66 ± 5	0.008
LV mean velocity of circumferential shortening (circ/sec)	1.18 ± 0.18	1.14 ± 0.16	0.075
Pulse doppler			
MV E wave velocity (cm/s)	100 ± 15	99 ± 17	0.81
MV A wave velocity (cm/s)	41 ± 9	42 ± 11	0.58
MV E/A ratio	2.5 ± 0.7	2.5 ± 0.7	0.75
MV A wave duration (msec)	122 ± 18	119 ± 20	0.36
MV deceleration time (msec)	154 ± 17	149 ± 20	0.075
Isovolumic relaxation time (msec)	76 ± 8	74 ± 7	0.038
Pulmonary vein S wave velocity (cm/s)	41 ± 10	43 ± 11	0.084
Pulmonary vein D wave velocity (cm/s)	61 ± 10	61 ± 12	0.89
LV myocardial performance index	0.30 ± 0.10	0.30 ± 0.09	0.82
LV stroke volume (ml)	70 ± 18	66 ± 17	0.72
LV cardiac output (l/min)	4.6 ± 1.2	4.4 ± 1.2	0.53
Tissue doppler			
MV lateral e’ wave tissue velocity (cm/s)	17.6 ± 2.6	18.6 ± 2.6	0.0003
MV lateral a’ wave tissue velocity (cm/s)	5.4 ± 1.1	5.9 ± 1.5	0.0003
MV lateral s’ wave tissue velocity (cm/s)	10.5 ± 1.8	11.1 ± 2.0	0.0005
MV E/lateral e’ ratio	5.8 ± 1.1	5.4 ± 1.0	0.001
MV septal e’ wave tissue velocity (cm/s)	13.9 ± 1.8	15.1 ± 2.2	0.0003
MV septal a’ wave tissue velocity (cm/s)	5.5 ± 1.0	5.9 ± 1.3	0.0008
MV septal s’ wave tissue velocity (cm/s)	8.3 ± 0.9	8.6 ± 0.9	0.001
MV E/septal e’ ratio	7.3 ± 1.2	6.7 ± 1.3	0.0003
Myocardial deformation			
LV mid circumferential strain (%)	−20.4 ± 2.3	−19.5 ± 1.7	0.0003
LV global longitudinal strain (%)	−19.0 ± 1.9	−19.8 ± 1.5	0.0003
Basal rotation (degrees)	−4.2 ± 2.4	−4.2 ± 2.0	0.89
Apical rotation (degrees)	6.2 ± 3.0	5.4 ± 2.5	0.009
Torsion (degrees)	10.4 ± 3.6	9.5 ± 3.0	0.026

Data are mean ± SD

*IVS* interventricular septal, *LV* left ventricular, *MV* mitral valve

* Adjusted for sex, age, BSA and for multiple comparisons

### Relationships of echocardiographic with clinical data

As shown in Table 3, age and blood pressure were the most common factors that contributed to differences in echocardiographic parameters between the T1D and control groups. In addition, body mass index (kg/m2) accounted in part for between group differences in left ventricular mean velocity of circumferential shortening and mitral valve lateral a’ wave tissue velocity. Low density lipoprotein (mmol/L) was also associated with between group differences in isovolumic relaxation time and mitral valve lateral s’ wave tissue velocity, and male gender was associated with between group differences for mitral valve lateral e’ wave tissue velocity and mitral valve E/lateral e’ ratio (Table 3).

**Table 3:** Determinants of differences in echocardiographic parameters in type 1 diabetes and control participants

	Beta estimate	p value
LV end-systolic dimension (cm)		
Age (years)	0.0564	0.0003
Body mass index (kg/m^2^)	0.0155	0.005
Male gender	0.1278	0.003
LV shortening fraction (%)		
Systolic blood pressure (mmHg)	0.105	0.006
Age (years)	−0.562	0.0015
LV ejection fraction (%)		
Systolic blood pressure (mmHg)	0.130	0.006
Age (years)	−0.752	0.0003
LV mean velocity of circumferential shortening (circ/sec)		
Age (years)	−0.016	0.015
Body mass index (kg/m^2^)	0.007	0.021
Isovolumic relaxation time (msec)		
Age (years)	0.995	0.002
Low density lipoprotein (mmol/L)	1.399	0.045
MV lateral e’ wave tissue velocity (cm/s)		
Systolic blood pressure (mmHg)	0.05489	0.015
Diastolic blood pressure (mmHg)	−0.1192	0.0003
Male gender	−0.8703	0.015
MV lateral a’ wave tissue velocity (cm/s)		
Age (years)	−0.09277	0.036
Body mass index (kg/m^2^)	0.04254	0.036
MV lateral S’ wave tissue velocity (cm/s)		
Systolic blood pressure (mmHg)	0.05823	0.0003
Diastolic blood pressure (mmHg)	−0.06427	0.003
Low density lipoprotein (mmol/L)	−0.3702	0.022
MV E/lateral e’ ratio		
Male gender	0.4664	0.003
MV septal e’ wave tissue velocity (cm/s)		
Systolic blood pressure (mmHg)	0.0395	0.015
Diastolic blood pressure (mmHg)	−0.067	0.003
Low density lipoprotein (mmol/L)	−0.531	0.004
Body mass index (kg/m^2^)	−0.0804	0.015
MV septal a’ wave tissue velocity (cm/s)		
Age (years)	0.0876	0.021
Body mass index (kg/m^2^)	0.0460	0.011
MV septal s’ wave tissue velocity (cm/s)		
Systolic blood pressure (mmHg)	0.034	0.0003
Diastolic blood pressure (mmHg)	−0.036	0.0003
Age (years)	−0.0778	0.016
Albumin to creatinine ratio (mg/mmol)	−0.0714	0.004
Low density lipoprotein (mmol/L)	−0.293	0.0006
Body mass index (kg/m^2^)	0.0289	0.041
MV E/septal e’ ratio		
Age (years)	−0.1276	0.011
LV mid circumferential strain (%)		
Systolic blood pressure (mmHg)	−0.0677	0.020
Age (years)	0.2991	0.021
Male gender	1.0854	0.019
LV global longitudinal strain (%)		
Age (years)	0.1788	0.011
Male gender	0.4800	0.041
Apical rotation (degrees)	N/A	N/A
Torsion (degrees)	N/A	N/A

*LV* left ventricular, *MV* mitral valve

## Discussion

During childhood, patients with T1D generally do not have clinical cardiovascular symptoms as there is a delay between the diagnosis of T1D and cardiovascular events which could be used for therapeutic interventions slowing down the progression of CV disease [8, 9, 29, 30]. Previous echocardiographic studies have shown variable differences in left ventricular (LV) geometry, mass and function [10–15]. Recent data have suggested that myocardial deformation imaging may be able to detect changes in myocardial mechanical properties prior to changes in ejection fraction in children and adolescents with T1D [16]. In addition, measures of early peripheral vascular dysfunction such as FMD and arterial stiffness have been associated with adverse long-term outcomes in adults with type 2 diabetes [31]. It is important to carefully characterize early changes in cardiac and peripheral vascular function in an adequately powered cohort of young patients with T1D to better understand factors that may predispose adverse long term outcomes in adulthood [6]. Our major findings were that compared to a matched group of healthy controls, adolescents with T1D exhibit: (1) higher blood pressure; (2) impaired endothelial function and increased arterial stiffness and; (3) altered myocardial velocities and strain.

Our first major observation was that we found increased systolic and diastolic blood pressures in adolescents with T1D compared with controls. Previous studies have consistently shown similar differences in blood pressure in children and young adults with T1D compared with controls [10, 32, 33]. The modest blood pressure elevation reported in otherwise healthy T1D cohorts would be silent clinically and has been previously reported to be associated with HbA1c [34], although we did not see such as relationship in this cohort (Spearman coefficient for HbA1c and SBP: −0.02, p = 0.75 and for DBP r = 0.11, p = 0.12). Nevertheless, similar blood pressure elevations within the “normal range” are important due to the increased risk of microalbuminuria and retinopathy associated with these changes [35, 36], as well as the potential impact on future cardiovascular risk [10, 32, 33, 37–39]. Furthermore, recent meta-analyses have suggested that even small reductions in blood pressure have a benefit on cardiovascular events, highlighting the potential importance of identifying and characterizing early changes in blood pressure, even within the normal range [40].

From a physiologic perspective, it seems unlikely that higher blood pressure in the T1D group was due to primary changes in cardiac function, since between group differences in mean heart rate, LV stroke volume and ejection fraction or cardiac output were very small. Instead, based on abnormalities described below, it seems more plausible that the higher blood pressure values may therefore have been due to an increase in peripheral vascular resistance, thereby leading to secondary echocardiographic changes. An increase in peripheral vascular resistance as the primary cause of early increases in blood pressure in T1D, rather than an increase in cardiac output, would be in contrast with early blood pressure changes in young, non-diabetic patients with essential hypertension. In this non-diabetic setting, increased cardiac output has been reported as the earliest hemodynamic change that precedes a rise in peripheral vascular resistance [41]. Fortunately, participants in the AdDIT study will be assessed longitudinally after the end of the initial study phase. As such, the natural history of early blood pressure abnormalities in patients with T1D will be determined in future follow up studies.

In addition to measuring blood pressure, to more fully evaluate the peripheral vasculature, we evaluated endothelial function with FMD and arterial stiffness using PWV [42], since peripheral vascular abnormalities have been associated with the development of clinical hypertension and future cardiovascular complications [31, 43, 44]. Previous studies have demonstrated abnormalities in vascular function in children with T1D, although these studies have, for the most part, been limited by small sample sizes [45], by differences in gender distributions [4, 46], by the lack of inclusion of HC [47], and by the inclusion of patients with T1D with children who have other conditions such as hypercholesterolemia [48]. Our second major observation was that in this well-defined, large cohort of adolescents, those with T1D exhibited impaired endothelial function with lower FMD and increased arterial stiffness as higher carotid-radial PWV. In contrast with a previous smaller study (n = 28), similar trends for carotid-femoral PWV did not reach significance [46]. Interestingly, in a multivariate analysis aimed at elucidating the specific factors underlying between group differences in vascular function, male gender had a negative impact on measures of endothelial function and arterial stiffness. In previous work, we and others have reported that adult women with T1D have higher arterial stiffness compared to men [49]. While sex hormone influences on autonomic function [50] and microvascular complications [51] have been reported elsewhere, this is to our knowledge, the first report documenting a potential role for gender as a modifier of arterial stiffness in adolescents with T1D. In the current analysis, although positive associations between PWV and BMI and PWV and blood pressure were perhaps expected [2], the association between male gender and higher PWV was surprising, and suggests that relationship gender and arterial stiffness changes in relation to pubertal status, a possibility that has been reported elsewhere and that requires further study [52]. Previous studies also have shown that in women FMD is higher when compared to men [53]. Less, however, is known about gender and FMD in adolescents. The current analysis suggests that as in adults, male gender has a negative effect on vascular function in adolescents. However, in our large cohort of patients with T1D and in contrast with previous work [46], the overall magnitude of the effect of T1D on arterial stiffness appears to be modest.

Our third major finding relates to changes in LV systolic and diastolic function in adolescents with T1D. Increased measures of LV systolic function by M-mode, such as shortening and ejection fraction as we found in our cohort, suggest that LV systolic function at rest is increased in adolescents with T1D. These observations have been suggested by others in a small (n = 39) adolescent cohort that only included boys, although the mechanism is not well understood [10]. Early changes in LV diastolic dysfunction found in our cohort, as indicated by longer isovolumic relaxation times, suggest differences in early relaxation and have been shown in previous small adolescent cohort studies [14, 15]. Similarly we found evidence by tissue Doppler assessment of changes in LV diastolic function with lower mitral valve lateral and septal myocardial velocities and higher mitral valve E/e’ ratios. Similar to observed changes in LV posterior wall thickness suggesting early LV hypertrophic changes, it is tempting to link changes in diastolic function to the higher blood pressure values within the “normal” range observed in this cohort. However, we recognize that other factors, such as activation of neurohormonal systems including the sympathetic nervous system or renin-angiotensin-aldosterone system, or deposition of advanced glycation end-products [54] may also be involved and could cause both higher blood pressure and ventricular remodeling.

In addition to these changes in M-mode echocardiography, we observed early changes in LV myocardial velocities and strain in adolescents with T1D compared with controls. We speculate again that the higher blood pressure values observed in this T1D cohort, although still within the “normal” range, may in part explain this ventricular remodeling. Alteration of LV longitudinal deformation with decreased global longitudinal strain, similar to what we found in our cohort, has been previously reported in children and adults with T1D [16, 55]. LV global longitudinal strain plays an important role in cardiac pump function and is primarily controlled by subendocardial longitudinal myofibres, which are more susceptible to afterload [16, 56]. Consequently, subclinical impairment in LV longitudinal deformation has been observed in the setting of many conditions associated with increased afterload or chronic pressure loading including hypertension and aortic stenosis [57, 58]. The increased LV mid circumferential strain we found may be an adaptive response to the decreased global longitudinal strain, as previously suggested in studies where radial and longitudinal contractility parameters have been measured in adults with T1D [55].

Our observations have potential clinical implications for the early identification of patients with T1D who are at high risk of future complications, and for earlier therapeutic interventions. FMD and arterial stiffness measures have been shown to predict future cardiovascular events in high risk groups, although the clinical utility of these parameters in early T1D has yet to be determined [31, 44]. The careful physiological phenotyping performed as part of our longitudinal observational study will therefore be important to determine if preclinical changes in cardiovascular function reported are associated with new onset hypertension, albuminuria, eGFR decline or even cardiovascular events in this cohort. Compared to previous cohorts involving adolescents with T1D, this dataset is unique, due to the longitudinal follow up that is planned in a subset of patients, and because findings form this observational, pilot cohort may be used to identify cardiovascular parameters of interest that could be modified by ACE inhibition/statin therapies being using in the separate cohort of participants currently enrolled in the AdDIT interventional study. On the therapeutic side, abnormalities in peripheral vascular function can be ameliorated with renal and cardiovascular protective and glucose lowering agents [26, 59]. Although the magnitude of the overall differences was small, it is potentially important to better understand early measures of cardiovascular dysfunction to potentially identify and treat high risk patients with T1D and thereby avoid end organ complications.

Our work has important limitations. Our adolescents with T1D were well matched in comparison to our controls, except for being heavier with consequent larger BSA and BMI. To account for these differences, we additionally reported models adjusted for sex, age and BSA. BSA was chosen, given that echocardiographic parameters are routinely indexed to this rather than BMI. Second, despite our relatively large sample size, we found only carotid-radial PWV and not carotid–femoral PWV differed between the two groups. Since carotid-femoral PWV is thought to reflect larger arteries compared to carotid-radial PWV, it is possible that early changes in arterial stiffness in adolescents with T1D are primarily limited to smaller arteries. Given the cross sectional design of this analysis, we were not able to determine the long term clinical significance of this and other statistically significant between group differences. Longitudinal data is being collected in this cohort as part of the AdDIT Observational Study and will help to clarify the relationships between these physiological measures and clinical outcomes. Finally, this study in adolescents was not designed to elucidate physiological mechanisms responsible for between group differences in cardiovascular function, such as early changes in nitric oxide bioavailability, autonomic tone, vitamin D levels or inflammatory biomarkers [60–63]. These important modulators of cardiovascular function should be further examined in adolescents in future work. Similarly, potential physiological factors that determine cardiovascular risk within the adolescent cohort such as the impact of glycemic variability—require further dedicated studies [64].

In conclusion, adolescents with T1D exhibit early changes in blood pressure, peripheral vascular function and left ventricular myocardial deformation indices with a shift from longitudinal to circumferential shortening. Longitudinal follow-up of these changes may allow early detection of those most at risk of early adult-onset cardiac disease that would preferentially benefit from preventive therapeutic interventions. Additional studies are also required to better elucidate subgroups of patients with early T1D with the most deleterious cardiovascular profiles, who may, as a consequence, be at the highest risk of future clinical disease.

## References

[1] Fang ZY, Prins JB, Marwick TH (2004). Diabetic cardiomyopathy: evidence, mechanisms, and therapeutic implications. Endocr Rev.

[2] Shah AS, Black S, Wadwa RP, Schmiege SJ, Fino NF, Talton JW (2015). Insulin sensitivity and arterial stiffness in youth with type 1 diabetes: the SEARCH CVD study. J Diabet Complications..

[3] Zarich SW, Nesto RW (1989). Diabetic cardiomyopathy. Am Heart J.

[4] Jarvisalo MJ, Raitakari M, Toikka JO, Putto-Laurila A, Rontu R, Laine S (2004). Endothelial dysfunction and increased arterial intima-media thickness in children with type 1 diabetes. Circulation.

[5] Laing SP, Swerdlow AJ, Slater SD, Botha JL, Burden AC, Waugh NR (1999). The British Diabetic Association Cohort Study, II: cause-specific mortality in patients with insulin-treated diabetes mellitus. Diabet Med.

[6] de Boer IH, Afkarian M, Rue TC, Cleary PA, Lachin JM, Molitch ME (2014). Renal outcomes in patients with type 1 diabetes and macroalbuminuria. J Am Soc Nephrol.

[7] Donahue RP, Orchard TJ (1992). Diabetes mellitus and macrovascular complications. An epidemiological perspective. Diabetes Care.

[8] Jarvisalo MJ, Putto-Laurila A, Jartti L, Lehtimaki T, Solakivi T, Ronnemaa T (2002). Carotid artery intima-media thickness in children with type 1 diabetes. Diabetes.

[9] Dalla Pozza R, Bechtold S, Bonfig W, Putzker S, Kozlik-Feldmann R, Netz H (2007). Age of onset of type 1 diabetes in children and carotid intima medial thickness. J Clin Endocrinol Metab.

[10] Kimball TR, Daniels SR, Khoury PR, Magnotti RA, Turner AM, Dolan LM (1994). Cardiovascular status in young patients with insulin-dependent diabetes mellitus. Circulation.

[11] Parikh A, Sochett EB, McCrindle BW, Dipchand A, Daneman A, Daneman D (2000). Carotid artery distensibility and cardiac function in adolescents with type 1 diabetes. J Pediatr.

[12] Gunczler P, Lanes R, Lopez E, Esaa S, Villarroel O, Revel-Chion R (2002). Cardiac mass and function, carotid artery intima-media thickness and lipoprotein (a) levels in children and adolescents with type 1 diabetes mellitus of short duration. J Pediatr Endocrinol Metab.

[13] Berger E, Sochett EB, Peirone A, Parikh A, Daneman D (2004). Cardiac and vascular function in adolescents and young adults with type 1 diabetes. Diabet Technol Ther.

[14] Wojcik M, Rudzinski A, Starzyk J (2010). Left ventricular diastolic dysfunction in adolescents with type 1 diabetes reflects the long- but not short-term metabolic control. J Pediatr Endocrinol Metab.

[15] Eltayeb AA, Ahmad FA, Sayed DM, Osama AM (2014). Subclinical vascular endothelial dysfunctions and myocardial changes with type 1 diabetes mellitus in children and adolescents. Pediatric Cardiol..

[16] Labombarda F, Leport M, Morello R, Ribault V, Kauffman D, Brouard J (2014). Longitudinal left ventricular strain impairment in type 1 diabetes children and adolescents: a 2D speckle strain imaging study. Diabet Metab..

[17] Adolescent type 1 Diabetes Cardio-renal Intervention Trial (AdDIT) Research Group (2009). Adolescent type 1 diabetes cardio-renal intervention trial (AdDIT). BMC Pediatr..

[18] Har RL, Reich HN, Scholey JW, Daneman D, Dunger DB, Moineddin R (2014). The urinary cytokine/chemokine signature of renal hyperfiltration in adolescents with type 1 diabetes. PLoS One.

[19] The fourth report on the diagnosis, evaluation, and treatment of high blood pressure in children and adolescents children and adolescents (2004). The fourth report on the diagnosis, evaluation, and treatment of high blood pressure in children and adolescents. Pediatrics.

[20] Schwartz GJ, Munoz A, Schneider MF, Mak RH, Kaskel F, Warady BA (2009). New equations to estimate GFR in children with CKD. J Am Soc Nephrol.

[21] Lopez L, Colan SD, Frommelt PC, Ensing GJ, Kendall K, Younoszai AK (2010). Recommendations for quantification methods during the performance of a pediatric echocardiogram: a report from the Pediatric Measurements Writing Group of the American Society of Echocardiography Pediatric and Congenital Heart Disease Council. J Am Soc Echocardiogr.

[22] Koopman LP, Slorach C, Hui W, Manlhiot C, McCrindle BW, Friedberg MK (2010). Comparison between different speckle tracking and color tissue Doppler techniques to measure global and regional myocardial deformation in children. J Am Soc Echocardiogr.

[23] Dragulescu A, Friedberg MK, Grosse-Wortmann L, Redington A, Mertens L (2014). Effect of chronic right ventricular volume overload on ventricular interaction in patients after tetralogy of fallot repair. J Am Soc Echocardiogr.

[24] Dallaire F, Slorach C, Hui W, Sarkola T, Friedberg MK, Bradley TJ (2015). Reference values for pulse wave Doppler and tissue Doppler imaging in pediatric echocardiography. Circ Cardiovasc Imaging..

[25] Cherney DZ, Miller JA, Scholey JW, Nasrallah R, Hebert RL, Dekker MG (2010). Renal hyperfiltration is a determinant of endothelial function responses to cyclooxygenase 2 inhibition in type 1 diabetes. Diabetes Care.

[26] Cherney DZ, Lai V, Scholey JW, Miller JA, Zinman B, Reich HN (2010). Effect of direct renin inhibition on renal hemodynamic function, arterial stiffness, and endothelial function in humans with uncomplicated type 1 diabetes: a pilot study. Diabetes Care.

[27] Kawano H, Motoyama T, Hirashima O, Hirai N, Miyao Y, Sakamoto T (1999). Hyperglycemia rapidly suppresses flow-mediated endothelium-dependent vasodilation of brachial artery. J Am Coll Cardiol.

[28] Cherney DZ, Scholey JW, Jiang S, Har R, Lai V, Sochett EB (2012). The effect of direct renin inhibition alone and in combination with ACE inhibition on endothelial function, arterial stiffness, and renal function in type 1 diabetes. Diabetes Care.

[29] Cho YH, Craig ME, Davis EA, Cotterill AM, Couper JJ, Cameron FJ (2015). Cardiac autonomic dysfunction is associated with high-risk albumin-to-creatinine ratio in young adolescents with type 1 diabetes in AdDIT (Adolescent type 1 diabetes cardio-renal interventional trial). Diabetes Care.

[30] Maftei O, Pena AS, Sullivan T, Jones TW, Donaghue KC, Cameron FJ (2014). Early atherosclerosis relates to urinary albumin excretion and cardiovascular risk factors in adolescents with type 1 diabetes: adolescent type 1 diabetes cardio-renal intervention trial (AdDIT). Diabetes Care.

[31] van Sloten TT, Schram MT, van den Hurk K, Dekker JM, Nijpels G, Henry RM (2014). Local stiffness of the carotid and femoral artery is associated with incident cardiovascular events and all-cause mortality: the Hoorn study. J Am Coll Cardiol.

[32] Pecis M, Azevedo MJ, Gross JL (1997). Glomerular hyperfiltration is associated with blood pressure abnormalities in normotensive normoalbuminuric IDDM patients. Diabetes Care.

[33] Yang GK, Maahs DM, Perkins BA, Cherney DZ (2013). Renal hyperfiltration and systemic blood pressure in patients with uncomplicated type 1 diabetes mellitus. PLoS One.

[34] Vilchez-Lopez FJ, Carral-Sanlaureano F, Coserria-Sanchez C, Nieto A, Jimenez S, Aguilar-Diosdado M (2011). Alterations in arterial pressure in patients with Type 1 diabetes are associated with long-term poor metabolic control and a more atherogenic lipid profile. J Endocrinol Invest.

[35] Imano E, Miyatsuka T, Motomura M, Kanda T, Matsuhisa M, Kajimoto Y (2001). Heart rate elevation and diabetic retinopathy in patients with type 2 diabetes mellitus and normoalbuminuria. Diabetes Res Clin Pract.

[36] Marcovecchio ML, Dalton RN, Schwarze CP, Prevost AT, Neil HA, Acerini CL (2009). Ambulatory blood pressure measurements are related to albumin excretion and are predictive for risk of microalbuminuria in young people with type 1 diabetes. Diabetologia.

[37] Schwab KO, Doerfer J, Krebs A, Krebs K, Schorb E, Hallermann K (2007). Early atherosclerosis in childhood type 1 diabetes: role of raised systolic blood pressure in the absence of dyslipidaemia. Eur J Pediatr.

[38] Maggio AB, Farpour-Lambert NJ, Montecucco F, Pelli G, Marchand LM, Schwitzgebel V (2012). Elevated E-selectin and diastolic blood pressure in diabetic children. Eur J Clin Invest.

[39] Urbina EM, Dabelea D, D’Agostino RB, Shah AS, Dolan LM, Hamman RF (2013). Effect of type 1 diabetes on carotid structure and function in adolescents and young adults: the SEARCH CVD study. Diabetes Care..

[40] Sundstrom J, Arima H, Jackson R, Turnbull F, Rahimi K, Chalmers J (2015). Effects of blood pressure reduction in mild hypertension: a systematic review and meta-analysis. Ann Intern Med.

[41] Lund-Johansen P (1991). Twenty-year follow-up of hemodynamics in essential hypertension during rest and exercise. Hypertension.

[42] Marcovecchio ML, Woodside J, Jones T, Daneman D, Neil A, Prevost T (2014). Adolescent Type 1 Diabetes cardio-renal intervention trial (AdDIT): urinary screening and baseline biochemical and cardiovascular assessments. Diabetes Care.

[43] de Andrade Junior CR, Silva EL, da Matta FM, Castier MB, Rosa ML, Gomes MB (2014). Influence of a family history of type 2 diabetes, demographic and clinical data on carotid intima-media thickness in patients with type 1 diabetes: a cross-sectional study. Cardiovasc Diabetol..

[44] Cardoso CR, Ferreira MT, Leite NC, Salles GF (2013). Prognostic impact of aortic stiffness in high-risk type 2 diabetic patients: the Rio deJaneiro type 2 diabetes cohort study. Diabetes Care.

[45] Babar GS, Zidan H, Widlansky ME, Das E, Hoffmann RG, Daoud M (2011). Impaired endothelial function in preadolescent children with type 1 diabetes. Diabetes Care.

[46] Stakos DA, Schuster DP, Sparks EA, Wooley CF, Osei K, Boudoulas H (2005). Cardiovascular effects of type 1 diabetes mellitus in children. Angiology..

[47] Wadwa RP, Urbina EM, Anderson AM, Hamman RF, Dolan LM, Rodriguez BL (2010). Measures of arterial stiffness in youth with type 1 and type 2 diabetes: the SEARCH for diabetes in youth study. Diabetes Care.

[48] Jarvisalo MJ, Lehtimaki T, Raitakari OT (2004). Determinants of arterial nitrate-mediated dilatation in children: role of oxidized low-density lipoprotein, endothelial function, and carotid intima-media thickness. Circulation.

[49] Ahlgren AR, Sundkvist G, Sandgren T, Lanne T (2002). Female gender increases stiffness of elastic but not of muscular arteries in type I diabetic patients. Clin Physiol Funct Imaging.

[50] Kim C, Pop-Busui R, Braffett B, Cleary PA, Bebu I, Wessells H (2015). Testosterone concentrations and cardiovascular autonomic neuropathy in men with type 1 diabetes in the epidemiology of diabetes interventions and complications study (EDIC). J Sex Med..

[51] Harjutsalo V, Maric-Bilkan C, Forsblom C, Groop PH (2015). Age at menarche and the risk of diabetic microvascular complications in patients with type 1 diabetes. Diabetologia.

[52] Shah AS, Dolan LM, Lauer A, Davis C, Dabelea D, Daniels SR (2012). Adiponectin and arterial stiffness in youth with type 1 diabetes: the SEARCH for diabetes in youth study. J Pediatr Endocrinol Metab.

[53] Har R, Lai V, Cherney D (2014). The effect of sex on endothelial function responses to clamped hyperglycemia in type 1 diabetes. Hypertens Res.

[54] Monnier VM, Sun W, Gao X, Sell DR, Cleary PA, Lachin JM (2015). Skin collagen advanced glycation end products (AGEs) and the long-term progression of sub-clinical cardiovascular disease in type 1 diabetes. Cardiovasc Diabetol..

[55] Fang ZY, Leano R, Marwick TH (2004). Relationship between longitudinal and radial contractility in subclinical diabetic heart disease. Clin Sci (Lond)..

[56] Lynch JJ, Ferro TJ, Blumenstock FA, Brockenauer AM, Malik AB (1990). Increased endothelial albumin permeability mediated by protein kinase C activation. J Clin Invest..

[57] Cikes M, Sutherland GR, Anderson LJ, Bijnens BH (2010). The role of echocardiographic deformation imaging in hypertrophic myopathies. Nat Rev Cardiol..

[58] Shah AM, Solomon SD (2012). Myocardial deformation imaging: current status and future directions. Circulation.

[59] Cherney DZ, Perkins BA, Soleymanlou N, Har R, Fagan N, Johansen OE (2014). The effect of empagliflozin on arterial stiffness and heart rate variability in subjects with uncomplicated type 1 diabetes mellitus. Cardiovasc Diabetol..

[60] Cherney DZ, Reich HN, Jiang S, Har R, Nasrallah R, Hebert RL (2012). Hyperfiltration and the effect of nitric oxide inhibition on renal and endothelial function in humans with uncomplicated type 1 diabetes mellitus. Am J Physiol Regul Integr Comp Physiol.

[61] Anaruma CP, Ferreira M, Jr., Sponton CH, Delbin MA, Zanesco A. Heart rate variability and plasma biomarkers in patients with type 1 diabetes mellitus: Effect of a bout of aerobic exercise. Diabetes Res Clin Pract. 2015. **(Epub ahead of print)**. 10.1016/j.diabres.2015.10.025 26678666

[62] Engelen L, Schalkwijk CG, Eussen SJ, Scheijen JL, Soedamah-Muthu SS, Chaturvedi N (2015). Low 25-hydroxyvitamin D2 and 25-hydroxyvitamin D3 levels are independently associated with macroalbuminuria, but not with retinopathy and macrovascular disease in type 1 diabetes: the EURODIAB prospective complications study. Cardiovasc Diabetol..

[63] Aguilera E, Serra-Planas E, Granada ML, Pellitero S, Reverter JL, Alonso N (2015). Relationship of YKL-40 and adiponectin and subclinical atherosclerosis in asymptomatic patients with type 1 diabetes mellitus from a European Mediterranean population. Cardiovasc Diabetol..

[64] Gorst C, Kwok CS, Aslam S, Buchan I, Kontopantelis E, Myint PK (2015). Long-term glycemic variability and risk of adverse outcomes: a systematic review and meta-analysis. Diabetes Care.

